# A Noble Profession: Nursing the Sick

**Published:** 1887-12-03

**Authors:** 


### A Noble Profession: Nursing the Sick.

### THE NURSING SISTERS OF ST. MARY, NEW KENT ROAD, S.E.

By a Member of the Sisterhood.

The first institution of these Sisters dates properly from the late Archbishop Tait, who, when Bishop of London, sanctioned their establishment, and their first work was amongst the sick poor in Bethnal Green. In January, 1864, Dr. Hume (the General Inspector of Hospitals at that time) sought their services for the Female Military Hospital at Portsea, which was placed, with the sanction of Lord William Paulet, under the charge of one of their community, assisted by a nurse. The success of this experiment led to an application, in January, 1867, from General Sir J. Yorke Scarlett, for a supply of nurses for the Soldiers' Wives' Hospital at Aldershot; and the sanction of Sir John Pakington, then Secretary of War, was given for the Nursing Sisters, who from that date were established with full authority in the camp.

It was a few years after this, in 1872, that the Nursing Sisters were in part brought back to London, their original field of work; and, true to their prestige, they started a emporary Home of Health for Invalid Women and Children at Hampstead, which has led to the more perfect and permanent institution now known as St. Osyth's Home of Health at Walton-on-Naze. This Home is especially for the recovery of workgirls broken down by hard work in London; and it is noteworthy that two wards for twelve free patients have been opened this year in honour of Her Majesty, and by Her Majesty's gracious command are especially named the "Victoria" and "Albert" wards. It is to be hoped that a second block may shortly be built by some rich friend of the poor. The estimated cost is £2,000, and the following are the trustees for the Home of Health: The Lord Egerton of Tatton, 7, St. James's Square, S.W.; Mr. G. A. Spottiswoode, 3, Cadogan Square, S.W.; and the Rev. G. Nugee, M.A., St. Austin's, New Kent Road. Any of the trustees will gladly receive and acknowledge donations towards the second block.

But it was in 1875 that the Sisters began their great work in South London, where a large mission field was opened to them in St. John's Parish, Walworth. Here it is that for some twelve years they have been ministering to the poor and sick in the most destitute neighbourhood of Locksfields, their attention being especially given to the poor machinist girls and seamstresses, whose sad state they were the first to discover and alleviate. They found that consumption and heart disease were rife amongst these children of toil, owing to their incessant work in factories, bad food, and miserable clothing. Indeed, so great was the evil that the Workgirls' Protection Society was founded, and at once placed under their charge, while the large property in the New Kent Road was assigned to them as a Central Home. This is now the well-known St. Mary's Home, where, amongst other things, girls are trained for domestic service, and also for nursing the sick, under the charge of Miss Ansell, the Lady Superior, who herself was trained for nursing in Guy's Hospital. It has been suggested that St. Mary's Home would form a most suitable centre for a South London Nursing Association, similar to that so successfully established by the Bishop of Bedford and his clergy for East London. Such an Association is now under consideration, with the advice and high approval of Dr. Steele, Superintendent of Guy's Hospital.

The plan of the South London Nursing Association is based, like that at the east end of London, upon a system of co-operation with the parochial clergy, each nurse being assisted by one or more ladies of charity. A large sum will be required for starting and maintaining so extensive a movement as that for providing South London with a nursing association upon an adequate basis, but many will doubtless give to so good a movement. Meanwhile, St. Mary's Home has established a department, with twelve beds, for the accommodation of the probationers who are engaged in nursing amongst the sick poor of South-East London. Contributions to this department are greatly needed, and will be gratefully received by Miss Ansell, Lady Superior, St. Mary's, New Kent Road.

